# Sex differences in vascular aging in response to testosterone

**DOI:** 10.1186/s13293-020-00294-8

**Published:** 2020-04-15

**Authors:** Kerrie L. Moreau, Matthew C. Babcock, Kerry L. Hildreth

**Affiliations:** 1 University of Colorado Anschutz Medical Campus, Bldg. L15 Rm 8111, 12631 East 17th Ave., PO Box 6511, Aurora, CO 80045 USA; 2 Denver Veterans Administration Medical Center, Geriatric Research Education and Clinical Center, Aurora, 80045 CO USA

**Keywords:** Vascular biology, Aging, Exercise, Estrogen, Testosterone

## Abstract

Large elastic arterial stiffening and endothelial dysfunction are phenotypic characteristics of vascular aging, a major risk factor for age-associated cardiovascular diseases. Compared to men, vascular aging in women appears to be slowed until menopause, whereafter vascular aging accelerates to match that seen in men. These sex differences in vascular aging have been attributed to changes in sex hormones that occur with aging. Although the role of estradiol in vascular aging in women has been highlighted in recent aging research, little is known about the impact of declining testosterone concentrations in both sexes. Importantly, while androgen concentrations generally decline with age in men, there are data that indicate reductions in androgen concentrations in women as well. Evidence suggests that low testosterone is associated with impaired endothelial function and increased arterial stiffness in men, although the effect of androgens on vascular aging in women remains unclear. Testosterone may modulate vascular aging by mitigating the effects of oxidative stress and inflammation, although there is sex specificity to this effect. The purpose of this review is to present and summarize the research regarding sex differences in vascular aging in response to androgens, specifically testosterone. Because exercise is a potent lifestyle factor for slowing and reversing vascular aging, we briefly summarize the available literature regarding the regulatory function of testosterone on vascular adaptations to exercise training.

## Introduction

Cardiovascular disease (CVD) is the leading cause of death globally, accounting for > 17.6 million deaths per year in 2016 and is projected to increase to > 23.6 million by 2030 [1]. By 2035, nearly half of the U.S. population is expected to have CVD, with associated costs of nearly $1.1 trillion [1]. This grim projection underscores the importance of understanding the pathobiology of CVD to inform effective interventions to prevent the onset of CVD.

Aging is the major risk factor for CVD, with 50% of adults aged 50–64 and 80% of adults ≥ 65 having some form of CVD [1]. In general, women have a lower prevalence of CVD until mid-life, whereafter prevalence rates increase exponentially and match those observed in men [1]. The reasons for the sex disparity in CVD are not completely understood but may be related to sex differences in vascular aging, a key mechanism in the etiology of age-associated CVD [2]. The phenotypic features of vascular aging include endothelial dysfunction and large elastic artery stiffening [2, 3]. Endothelial dysfunction is characterized by reduced endothelial-dependent vasodilation, due in large part to a decrease in the bioavailability of nitric oxide (NO) [4]. The decrease in endothelial-dependent vasodilation can lead to increased vascular smooth muscle cell tone and contribute to large elastic arterial stiffening [3]. Other structural changes in the arterial wall that contribute to arterial stiffening include elastin degradation and increased collagen deposition, and crosslinking of elastin and collagen by advanced glycation end-products [5].

Sex differences in the rate of vascular aging have been attributed to differences in gonadal aging and changes in sex hormones in women and men. To date, most of the available literature regarding the modulatory influence of sex hormones on vascular aging has focused on the effects of menopause and declines in estradiol in women; there has been little discussion on the impact of testosterone. Some data suggest that declining testosterone levels in men contribute to accelerated vascular aging, although the effects in women remain equivocal. In this review, we will discuss sex differences in the modulatory influence of androgens, specifically testosterone, on vascular aging, and the potential influence of testosterone on vascular adaptations to regular exercise. It is outside the scope of the review to discuss other androgenic hormones (e.g., dehydroepiandrosterone [DHEA], androstenedione), conditions associated with androgen excess (e.g., polycystic ovary syndrome [PCOS]), or effects in transgender individuals.

## Quantification of testosterone

Considerable heterogeneity exists among quantification and reference standards of testosterone. Testosterone concentrations exhibit significant diurnal and day-to-day variation; measurement in the morning under fasted conditions on at least two separate occasions is therefore recommended [6]. Further variability in the reporting of testosterone concentrations is introduced through the use of different analytic methods. The measurement of total testosterone is commonly accomplished via direct assay (radioimmunoassay, enzyme-linked immunosorbent assay, or chemiluminescent immunoassay). Although these methods are technically simple, rapid, and relatively inexpensive, their accuracy is limited, especially in lower testosterone ranges (< 300 ng/dL), and testosterone concentrations are frequently overestimated [7]. Accurate measurements, especially in individuals with very low testosterone concentrations (as low as 2 ng/dL), require the use of liquid chromatography/tandem mass spectrometry (LC/MS-MS). While LC/MS-MS yields highly accurate results, it is relatively expensive and time-intensive [7].

Total testosterone includes both free and bound fractions. Because the majority of circulating testosterone is tightly bound to sex hormone-binding globulin (SHBG), measures of testosterone that quantify bioavailable hormone—the fraction that is either free or loosely bound to other proteins, mainly albumin—are frequently reported. Free testosterone, which accounts for 1–4% of total testosterone [8] can be measured using equilibrium dialysis or ultrafiltration; however, it is more commonly calculated from concentrations of total testosterone, SHBG, and albumin [9–11]. Bioavailable testosterone, which includes free testosterone plus the fraction that is loosely bound to albumin, is also calculated from concentrations of total testosterone, SHBG, and albumin.

## Testosterone changes with aging

### Women

Understanding changes in androgens across the lifespan in women is challenging, due to both their complex physiology and the use of different assays between studies, some of which are unreliable in the normal female range. Reference ranges for testosterone in women have yet to be established; however, reported testosterone concentrations in small cohorts of young women tend to range from 35–50 ng/dL [12, 13]. The sources of androgens in women include the adrenals and ovaries, as well as substantial contributions from the conversion of prohormones in peripheral tissues [14].

Although there are wide variations in androgen levels in healthy women across the lifespan, all androgens and androgenic prohormones including testosterone, appear to decline with age [12]. In contrast to estradiol, most evidence to date suggests these declines are due to age rather than to menopause per se. For example, in a cross-sectional study of women aged 18–75, total testosterone and mean free testosterone levels among those age ≥ 65 were 55% and 49% lower, respectively, compared to women aged 18–24 [12]. Interestingly, the steepest drop in testosterone levels occurred in the early reproductive years, with little to no further decrease in mid- and later life. Additionally, among women aged 45–54, there were no differences in testosterone levels between pre- and postmenopausal women [12]. These data are supported by a prospective longitudinal study of mid-life women followed annually for 7 years through the menopause transition [15]. Mean testosterone levels did not change over the study period and were unrelated to either age or menopause, perhaps due to the limited age range in the study (46–62 years) [15]. Testosterone levels have been noted by others to be maintained after age 65 as well [16].

Ovarian production of testosterone after menopause has been debated. Oophorectomized women have lower levels of total and free testosterone compared to non-oophorectomized women [12], with decreases in testosterone of approximately 50% following oophorectomy in postmenopausal women [17]. Others, however, suggest that the sole source of testosterone after menopause is via conversion of DHEA [18].

### Men

It is well established that testosterone declines by approximately 1% per year in men after the third decade [19]. Approximately 20% of men age ≥ 60 and 50% of men age ≥ 80 have serum testosterone levels below the normal range for young men [20]. Declines in bioavailable testosterone are even greater than declines in total testosterone, as measurements of total testosterone include the fraction that is tightly bound to SHBG, which increases with age [20]. Gradual declines in serum testosterone with aging are accompanied by modest increases in follicle-stimulating hormone (FSH) and luteinizing hormone (LH), which often remain within the normal range [19]. While the occurrence of low testosterone without symptoms does not meet the definition of “androgen deficiency” set by the Endocrine Society [6], total testosterone < 300 ng/dL has historically been used as a cutoff to define low testosterone. Recent studies using mass spectroscopy have established slightly higher reference ranges of 11 nmol/L (317 ng/dL) [21] and 12 nmol/L (348 ng/dL) [22]. Free testosterone concentrations may provide additional insight, especially in men with conditions that affect SHBG (e.g., obesity, type 2 diabetes mellitus) and reference ranges of 64 pg/mL [21] and 70 pg/mL [22] have been established.

Age-related declines in testosterone in men reflect impairments in both testis function and hypothalamic regulation of gonadotropin secretion. The number of testosterone-producing Leydig cells is decreased in older men; in addition, the circadian variation in testosterone levels observed in younger men, with peak levels in the early morning, is attenuated in older men, suggesting impairment of the normal pulsatile gonadotropin-releasing hormone (GnRH) secretion [19]. Age-related changes in dihydrotestosterone (DHT), the more potent metabolite of testosterone produced primarily via conversion of testosterone by 5-alpha-reductase in peripheral tissues, are less clear. Both decreases and increases in DHT have been reported [23, 24]; all things considered, declines in DHT with age appear to be less pronounced than declines in testosterone [25]. Furthermore, the significance of circulating levels of DHT is questionable given the difficulty of measurement at the tissue level, where the vast majority of DHT production and action occurs.

## Testosterone and CVD

### Women

The observational data with regard to endogenous testosterone and CVD morbidity and mortality in women are mixed, with some showing positive [26–29], negative [30, 31], or no association [32–34]. Discrepancies in the literature may be related to differences in study designs (e.g., cohort vs nested case-control, varying follow-up periods), population (e.g., younger vs older women), androgen assays, and/or assessment of CVD (e.g., medical records, self-report). It is possible that an optimal range of testosterone may exist for cardiovascular risk. For example, in a prospective, population-based study of postmenopausal women aged 50–91 years, *low* total testosterone and *high* bioavailable testosterone were independently associated with increased risk of incident CVD events [35]. Women in the lowest quintile for total testosterone had a 62% increased risk of a first-ever CVD event compared to women at all other quintiles, independent of age, adiposity, lifestyle, and menopause status, suggesting a potential threshold level [35]. Somewhat paradoxically, postmenopausal women with the lowest total testosterone levels were younger and had more favorable CVD risk factor profiles (e.g., lower body mass index and blood pressure), although they also had lower HDL cholesterol and higher triglyceride levels. However, the association between low total testosterone and increased CVD events was attenuated after eliminating events within the first 5 years of follow-up, suggesting that low total testosterone could be a marker of subclinical CVD. In this regard, postmenopausal women with lower testosterone levels were found to have greater carotid artery intimal-medial thickening, a biomarker of subclinical atherosclerosis [36]. In contrast to total testosterone, levels of bioavailable testosterone in the highest quintile were associated with an increase in incident CVD events. Levels of total and bioavailable testosterone were not concordant, and women with high bioavailable testosterone had less favorable CVD risk profiles (e.g., hypertension, diabetes, metabolic syndrome). Adjusting for these factors attenuated the association between bioavailable testosterone and CVD events; thus, it is not clear whether bioavailable testosterone is a marker or mediator of CVD risk in women.

It is possible that the ratio of testosterone to estradiol or a higher free androgen index (testosterone/SHBG; FAI) due to a decline in SHBG, rather than testosterone, may be important for postmenopausal CVD risk. In a 12-year follow-up study of postmenopausal women, a higher testosterone/estradiol ratio was associated with increased risk for CVD, CHD, and heart failure events, independent of age, hormone therapy, and traditional risk factors [26]. Similarly, higher FAI and lower SHBG were associated with increased risk for CVD in postmenopausal women who were not using hormone therapy; however, this was not independent of body composition or CVD risk factors [27].

Whether exogenous testosterone therapy infers CVD risk or harm in women is unclear. There have been no randomized controlled trials examining the effects of testosterone treatment on CVD in women. Although CVD outcomes per se were not assessed, a small randomized controlled trial in elderly women with heart failure found that 6 months of testosterone treatment was safe and effective for improving functional capacity, insulin resistance, and muscle strength [37]. Nonetheless, the only evidence-based indication for testosterone therapy for women is for the treatment of hypoactive sexual desire disorder (HSDD); there is insufficient evidence to support the use of testosterone for the treatment or prevention of any other symptom or clinical condition [16].

### Men

In contrast to women, observational studies in men have consistently reported an association between low testosterone and increased CVD risk and mortality [38–46]. In a recent 18-year follow-up study of men aged 30 and older, higher mortality rates were observed in men who demonstrated the most pronounced age-related decline in total testosterone, independent of age, baseline testosterone level, and lifestyle factors [47]. However, studies suggesting possible cardiovascular harm in older men receiving testosterone therapy have cautioned the use of testosterone in older men [48–51]. A randomized controlled trial conducted in frail older men with baseline testosterone levels between 100 and 350 ng/d was terminated early because of increased rates of CVD events in men randomized to testosterone than controls [50]. In a retrospective cohort study, male veterans with low testosterone (< 300 ng/dL) who initiated testosterone therapy ~ 1.5 years following coronary angiography had greater mortality and cardiovascular events than those who were not taking testosterone [49]. Because these studies included men with pre-existing CVD, it is plausible that cardiovascular risk is diminished in healthy men. In this regard, we previously reported no increase in adverse cardiovascular events in healthy men with borderline low to normal testosterone treated with testosterone compared to placebo [52]. Importantly, there have been no large, long-term, placebo-controlled randomized controlled trials of testosterone therapy on cardiovascular outcomes to provide any definitive conclusions about CVD risk with testosterone in men [40].

## Testosterone effects on vascular aging

### Endothelial dysfunction

Endothelial dysfunction, characterized by reduced endothelial-dependent vasodilation, is a significant predictor of cardiovascular events [53]. Because the vascular endothelium plays a key role in the maintenance of vascular health [54], the loss of normal endothelial function is believed to be a critical step in the initiation and progression of atherosclerosis [2]. Endothelial function is commonly assessed using the non-invasive technique of brachial artery flow-mediated dilation (FMD), a measure of macrovascular endothelial function. Microvascular endothelial function can be assessed by digital pulse amplitude reactive hyperemia using peripheral arterial tonometry (i.e., Endopat) and pharmacologically by administering endothelial-dependent agents such as acetylcholine into the brachial artery and measuring the forearm blood flow response. Importantly, brachial artery FMD, digital peripheral arterial tonometry, and forearm blood flow to acetylcholine are predictors of future CVD events demonstrating their utility for assessing CVD risk and preclinical disease [55, 56].

Aging is associated with a progressive decline in both macro- and microvascular endothelial function even in apparently healthy adults; however, sex differences exist in the rate of this decline. Men demonstrate a gradual decline after the fourth decade, and women approximately a decade later, whereafter it declines much more rapidly, specifically in postmenopausal women [57, 58]. This sex difference in the age-related impairment in endothelial function has been attributed to the decline in estrogens with menopause in women. Whether androgens contribute to the age-related endothelial dysfunction in women and men is not clear.

#### Women

The aging process in women is unique because of the influence of both gonadal and chronological aging. The menopause transition (i.e., perimenopause) intersects with aging, typically beginning in the mid-to-late 40s, and lasting several years before the final menstrual period [59]. During this time, profound changes in sex hormones, CVD risk factors, and symptoms (i.e., hot flashes, sleep disturbances, depression) occur. There are two stages to the menopause transition: early perimenopause (menstrual cycle changes of ≥ 7 days) and late perimenopause (≥ 2 months of amenorrhea). We demonstrated that although the onset of endothelial dysfunction appears to begin during early perimenopause in healthy women, the late perimenopausal transition seemed to be the most critical time period for adverse changes in the vasculature, consistent with previous observations [60–62]. Endothelial function, measured via brachial artery FMD, was reduced in early perimenopausal compared to premenopausal women; however, the level of impairment in late perimenopausal (~ 34%) was twice that of the age-matched early perimenopausal women (~ 17%) [63]. These differences were independent of age and CVD risk factors. The reduction in FMD was strongly correlated with higher FSH and lower estradiol levels, but there was no correlation with testosterone. It is possible that the immunoassay measurement of testosterone used in our study lacked the sensitivity and precision to accurately measure testosterone (accuracy of 17 ng/dL), and a more sensitive and specific assay such as LC-MS/MS may show a relation between testosterone and FMD.

In this regard, in a recent study that used LC-MS/MS to measure sex hormones, Thurston et al. showed that high levels of free testosterone (and low SHBG) were associated with reduced brachial artery FMD in women aged 40–60 years [64]. These data are consistent with previous observations in postmenopausal women showing FAI was an independent predictor of changes in endothelial function, where a higher baseline FAI was associated with a greater reduction in FMD after 29 months of follow-up [65]. However, in contrast, other studies have shown that low endogenous testosterone was associated with reduced endothelial function measured via FMD in healthy postmenopausal women [66] and by plethysmography in oophorectomized early postmenopausal women taking hormone therapy [67].

Both age and menopause stage may modulate the association between testosterone and endothelial function. In the Multi-Ethnic Study of Atherosclerosis (MESA), higher testosterone levels (and low SHBG) were associated with worse FMD in postmenopausal women age < 65, but not in those age ≥ 65 [68]. Additionally, in the study by Thurston et al., the association between testosterone and FMD was modified by menopause stage, in that higher levels of total testosterone were associated with a more favorable FMD in perimenopausal women [64]. However, the authors urged caution in the interpretation of these data due to the small number of perimenopausal women.

To our knowledge, there is only one study that has examined the effect of exogenous testosterone on endothelial function in healthy women. Worboys et al. demonstrated that 50 mg of parenteral testosterone increased brachial artery FMD and nitroglycerine-mediated vasodilation, a measure of smooth muscle cell function, after 6 weeks in healthy postmenopausal women who were chronically using estradiol therapy for > 6 months [69].

#### Men

Unlike the available evidence that links endothelial dysfunction with gonadal aging and the menopause transition in women, it is unclear whether endothelial dysfunction occurs with age-associated declines in testosterone in men in the absence of disease. Cross-sectional studies have shown that low serum testosterone is associated with both reduced [70–73] and higher [74] macro- and microvascular endothelial function in men of various ages. In those studies that have shown that low total and free testosterone were associated with impaired endothelial function, measured by FMD and by digital peripheral tonometry, in men over a broad age range, adjustments for age, body mass index, and CVD risk factors did not abolish the associations [70–72]. Similarly, brachial artery FMD and nitroglycerine-mediated vasodilation were moderately reduced in middle-aged and older men with end-stage kidney disease who were classified as androgen-deficient (testosterone < 300 ng/dL) compared to those without androgen deficiency [73]. Moreover, there were strong positive correlations between brachial artery FMD and nitroglycerine-mediated vasodilation with total and free testosterone concentrations in the patients classified as androgen-deficient [73].

In contrast, in middle-age and older men with late-onset hypogonadism, lower testosterone levels were correlated with higher brachial artery FMD, albeit the correlation was weak [74]. These data are consistent with studies conducted in men undergoing androgen deprivation treatment (ADT) by either surgical or chemical castration for prostate cancer. In middle-age/older men who had either surgical or chemical castration for at least 6 months, endothelial function measured by FMD was higher than age-matched healthy controls and controls who were in remission from a non-prostate malignancy [75]. Moreover, the levels of total and free testosterone were inversely correlated with brachial artery FMD [75]. Similarly, endothelial function improved in older men following 3 months of ADT [76]. However, these data are inconsistent with a case-control study that showed that brachial artery FMD was reduced in older men with prostate cancer treated with ADT compared to men without prostate cancer that were matched for age, physical activity, and comorbid CVD and body composition [77]. Discrepancies in the literature could be explained by differences in participant characteristics (age, presence/absence of comorbidities), length of androgen deficiency, and/or in the methodological assessment of FMD, including occlusion placement (upper arm vs lower arm occlusion), and in the analysis of the peak FMD response post cuff deflation (i.e., pre-determined time point vs. continuous). Upper arm occlusion models are considered a less valid method for assessment of endothelial function because they are confounded by ischemia compared to models using lower arm occlusion [78]. Moreover, using post-deflation pre-determined time points as the measure for peak FMD (e.g., 50–60 s) may underestimate the true FMD response leading to misleading conclusions regarding group differences [79].

Similar to the cross-sectional studies, the data are inconsistent with regard to the effects of exogenous testosterone and endothelial function in men. In a recent meta-analysis that examined the available literature on the effect of testosterone treatment on endothelial function measured by brachial artery FMD in hypogonadal men, there was overall no significant benefit or harm; however, there was high heterogeneity in the response to treatment [80]. In addition to methodological differences, it is plausible that the differential effects of testosterone on endothelial function could be related to the type of formulation and/or length of treatment, age, and/or study population. For example, oral and transdermal testosterone formulations improved macro- and microvascular endothelial function [81–86], whereas those that administered testosterone intramuscularly showed impairments in endothelial function [87–89]. Additionally, the studies that showed improvements in endothelial function with testosterone treatment were conducted in middle-aged and older men [81–86], whereas those that reported impairments in endothelial function following testosterone treatment were conducted in younger hypogonadal men [87–89]. In the studies conducted in younger men, the causes of hypogonadism were likely related to Klinefelter syndrome, Leydig cell insufficiency, and or hypothalamic-pituitary insufficiency, and thus, these men may respond differently to testosterone treatment than middle-age and older men who have declines in testosterone secondary to aging.

### Large elastic arterial stiffening

Stiffening of the large elastic arteries is an independent risk factor for age-associated CVD in both women and men [90, 91]. Large elastic arteries exert a powerful cushioning function as they buffer the rise in systolic pressure by storing a portion of the ejected stroke volume during systole and delivering nearly steady blood flow to perfuse tissues and organs. However, age- and/or disease-related arterial stiffening interferes with the ability of the arteries to dampen the intermittent flow and pressure generated by the left ventricle, leading to increases in systolic and pulse pressures, left ventricular hypertrophy, atherosclerotic disease, congestive heart failure, and greater pulsatile flow to the microvasculature of target organs, particularly those requiring high blood flow and low arteriolar resistance, such as the brain, kidney, and eye [91, 92]. The gold standard for measuring large elastic arterial stiffness is aortic pulse wave velocity (PWV) which is an indicator of the speed of the pulse wave generated by left ventricular contraction through the aorta. More specifically, it is assessed by measuring the pulse transit time and the distance traveled by the pulse between the carotid and femoral arteries (cf-PWV), where higher values indicate greater stiffness. Arterial stiffness can also be measured directly using a combination of imaging of carotid artery diameter changes with systole and diastole and changes in central arterial pressure (i.e., pulse pressure).

Large elastic artery stiffness increases progressively in both women and men, but there are disparities in the literature on whether the rate of arterial stiffening with aging differs by sex. Cross-sectional studies reported that the progression in the age-related increase in cf-PWV appears to be similar between women and men [93–95]. In contrast, 9-year follow-up data from the Baltimore Longitudinal Study of Aging showed that although there was no overall sex difference in PWV when the full age spectrum was considered, sex differences emerged in the rate of increase in PWV with aging, with steeper increases in men compared to women after the age of 50 [96]. In studies using measures that are sensitive to artery diameter, women have greater age-related rates of aortic and carotid artery stiffening compared to men, likely reflecting intrinsic differences in geometry [95, 97]. Although sex differences in the rate of age-related arterial stiffening may be methodology-dependent, the contributions of changes in gonadal hormones with menopause and andropause are unclear and warrant further study.

#### Women

There is evidence to suggest that the menopause transition and changes in gonadal hormones contribute to increased large elastic arterial stiffness with aging in women. Several studies demonstrated that the increase in arterial stiffness was more rapid after menopause [98–100]. Our work showed that the reduction in carotid artery compliance (increased arterial stiffness) was more rapid during perimenopause, particularly the late perimenopausal transition. Despite being the same age, the difference in carotid artery compliance between late perimenopausal and premenopausal was much larger than the difference observed between early perimenopausal and premenopausal women [101]. After adjusting for age, the effect of menopause stage was no longer significant, consistent with others that reported no difference in PWV across menopausal transition stages after adjusting for age, smoking, and systolic blood pressure [102]. However, because age and menopausal stage are highly correlated, it is difficult to uncouple the tight association between these two factors. It is likely that both aging and changes in sex hormones with the menopause transition contribute to large artery stiffening.

In our cross-sectional study, we reported weak to moderate correlations between carotid artery compliance and the sex hormones FSH, estradiol, estrone, and progesterone, whereas the correlation with testosterone did not reach statistical significance (*r* = 0.18, *P* = 0.08) [101]. These data are consistent with other observations showing no correlation between total testosterone and measures of arterial stiffness [65, 103, 104]. In contrast to total testosterone, FAI has been shown to be associated with large elastic arterial stiffening. In postmenopausal women, FAI was an independent predictor of PWV [65, 103, 104] and predicted the increase in PWV independent of age and blood pressure [65]. To our knowledge, whether testosterone treatment in postmenopausal women contributes to large elastic arterial stiffening has not been studied.

#### Men

To date, most studies examining the association between testosterone and arterial stiffness have been conducted in specific populations, including hypogonadal men [105, 106], men with type 2 diabetes [107], and male hemodialysis patients [108]. However, several studies in more general populations of men also support an association between lower levels of testosterone and increased arterial stiffness. Dockery et al. performed a secondary analysis in a mixed group of older men (healthy volunteers, men with untreated hypertension, and men with prostate cancer prior to ADT) and found an inverse relation between the free testosterone index (FTI) and PWV in both the overall study population and in the subgroup of men with no CVD or use of vasoactive medications [109]. These data are consistent with other observations that reported a strong association between total testosterone and measures of arterial stiffness (i.e., PWV and augmentation index) in men without CVD [72, 110]. Interestingly, this association was especially prominent among younger men (age < 60) and among those with higher blood pressure (mean pressure ≥ 102 mmHg) [110]. In a longitudinal study, testosterone was negatively correlated with carotid arterial stiffness and predicted the arterial stiffness index, even after adjusting for age, pulse pressure, body mass index, glucose, and total cholesterol [111].

Several intervention studies also support an association between testosterone and increased arterial stiffness. Studies in men with prostate cancer have consistently demonstrated increased arterial stiffness with the initiation of ADT that is reversed with the cessation of treatment [112–114]. In general, testosterone intervention studies have shown decreased arterial stiffness. In a study conducted in older men with untreated hypogonadotropic hypogonadism who had higher PWV than age and weight-matched controls, PWV decreased by ~ 7% at 48 h after initiating transdermal testosterone treatment and remained stable with continued treatment at 3 months [106]. Although the improvement in PWV was significant, testosterone treatment did not decrease arterial stiffness to that of controls [106]. In obese men with severe sleep apnea, 18 weeks of intramuscular testosterone undecanoate treatment was also shown to decrease arterial stiffness measured by augmentation index compared to placebo [115]. In men with coronary heart disease and low testosterone (≤ 12 nmol/L [~ 345 ng/dL]), 8 weeks of oral testosterone undecanoate decreased augmentation index compared to placebo treatment [116]. Finally, although we did not find an overall effect of testosterone on carotid artery stiffness different from placebo, in exploratory analyses of within-group changes, we found carotid arterial compliance (inverse of stiffness) was significantly increased after 12 months in healthy older men treated to usual-range, as opposed to lower-range testosterone [85].

## Potential biological mechanisms associated with testosterone effects on vascular aging

**Fig. 1:**
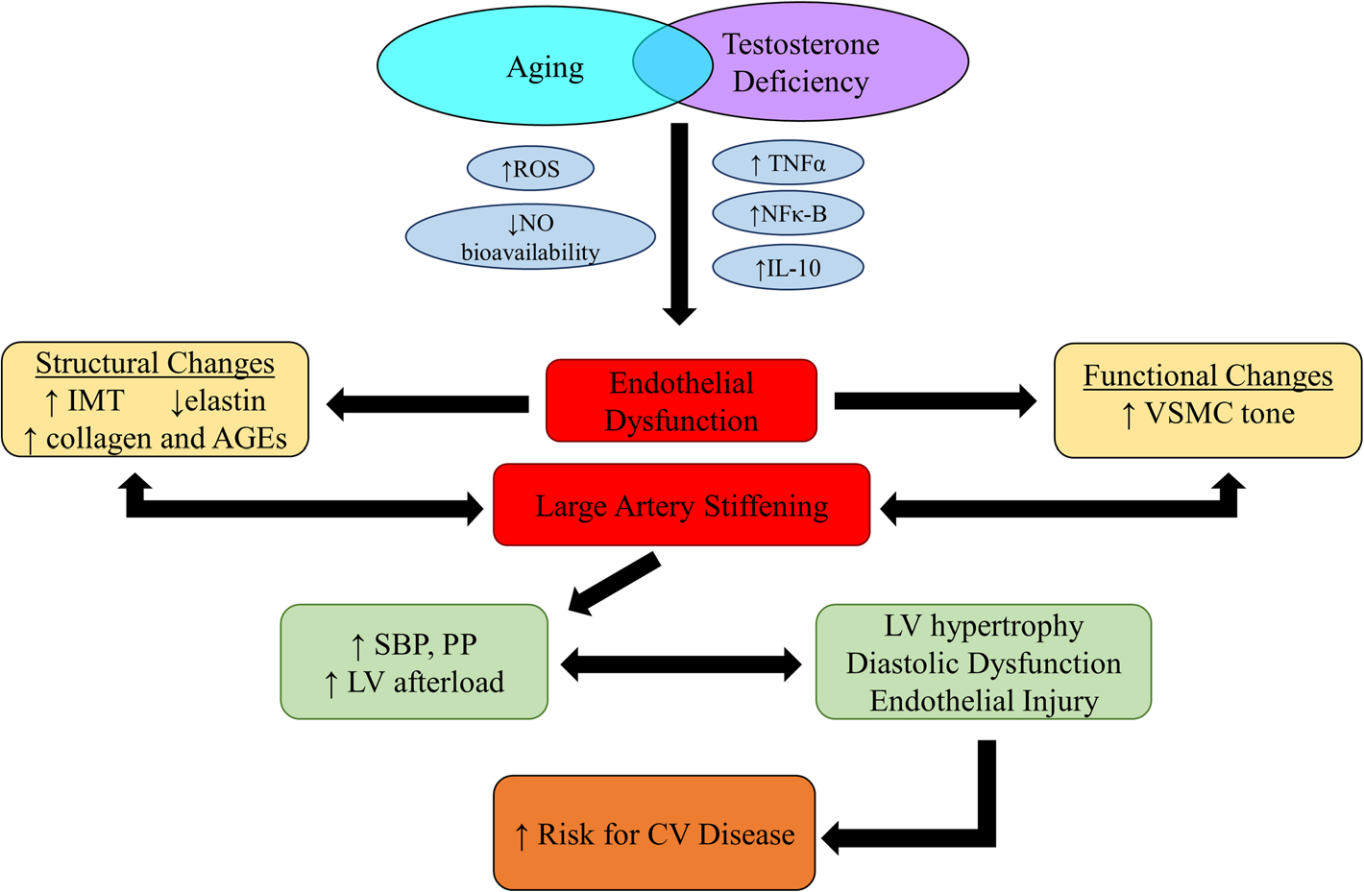
Hypothesized mechanisms by which testosterone deficiency may contribute to vascular aging in women and men. AGEs, advanced glycation end products; CV, cardiovascular; IL-10, interleukin-10; IMT, intima-media thickness; LV, left ventricle; NFκ-B, nuclear factor κ-B; NO, nitric oxide; PP, pulse pressure; SBP, systolic blood pressure; ROS, reactive oxygen species; TNFα, tumor necrosis factor-α, VSMC, vascular smooth muscle cell

Our working hypothesis for the mechanisms by which testosterone deficiency contributes to vascular aging in women and men is displayed in Fig. 1. Key mechanisms that contribute to the vascular aging process in women and men include oxidative stress and inflammation. Oxidative stress represents the imbalance between the generation and destruction of reactive oxygen species within multiple cellular compartments including the plasma membrane (e.g., NADPH oxidase), cytoplasm (e.g., xanthine oxidase), mitochondria (e.g., electron transport chain and oxidative phosphorylation), and peroxisomes (e.g., lipid oxidation). Excessive reactive oxygen species generation and inflammatory mediators (e.g., tumor necrosis factor[TNF]-α, nuclear factor κ-B) impair endothelial function and increase large elastic arterial stiffness by suppressing NO production by impairing the function of endothelial nitric oxide synthase (eNOS), the enzyme that synthesizes NO from the substrate l-arginine, and by reacting with NO that is released, decreasing its overall bioavailability. Reactive oxygen species and inflammatory mediators also contribute to arterial stiffening by degrading elastin and increasing the deposition of collagen and calcium and by causing greater crosslinking of elastin and collagen by advanced glycation end-products. Although there has been extensive research on the mechanisms by which estrogen deficiency associated with the menopause transition contributes to vascular aging in women, mechanistic insight on the modulatory influence of testosterone on vascular aging in women and men is lacking.

Testosterone has direct actions on the vascular wall via androgen receptors or by metabolism to DHT and indirect effects via aromatization to estradiol and its metabolites. Similar to estradiol, testosterone induces rapid vasodilation through both non-genomic endothelium-dependent and independent (i.e., direct effects on arterial smooth muscle cells) effects. The endothelium-dependent effects of testosterone are due to increased NO production via androgen receptor-mediated activation of eNOS and to the release of factors from the endothelium that cause hyperpolarization and relaxation of vascular smooth muscle cells [117–119]. The endothelium-independent effects include the modulation of calcium influx and activation of potassium channels in smooth muscle cells [83, 84, 120].

Whether testosterone influences the vascular aging process by modulating oxidative stress and inflammation is unknown. In males, most of the available evidence suggests that androgen deficiency is associated with elevated oxidative stress and inflammation and that testosterone has both antioxidant and anti-inflammatory properties. Male rats treated with testosterone showed a two-fold increase in the activity of the antioxidants catalase and superoxide dismutase [121], and orchiectomized male rats had a reduced antioxidant defense system and elevated lipid peroxidation and nitrotyrosine (oxidatively modified amino acid) in the left ventricle that was associated with reduced left ventricular contractility; testosterone treatment ameliorated the pro-oxidant state and increased contractility [122, 123].

In studies conducted in humans, testosterone concentrations were inversely correlated with oxidized low-density lipoprotein and pro-inflammatory cytokines in older men [124, 125]. Testosterone supplementation decreased TNF-α and increased the anti-inflammatory cytokine interleukin-10 in hypogonadal older men [125]; however, inflammatory markers (C-reactive protein, interleukin-6) did not change following 1 year of testosterone treatment in obese older men with comorbid conditions who had low testosterone [126]. In young men, TNF-α and interleukin-6 concentrations increased the following gonadal hormone suppression with GnRH agonist and an aromatase inhibitor [127]. With the exception of IL-6 concentrations, these increases were nearly abrogated with testosterone add-back therapy [127].

In contrast, evidence in females suggests that testosterone could be associated with increased oxidative stress and inflammation. Aortic endothelium-dependent vasodilation was increased in ovariectomized (OVX) spontaneously hypertensive rats treated with conjugated equine estrogen (CEE) compared to OVX rats, but was abolished in OVX rats treated with CEE plus testosterone due to increased superoxide production through activation of NADPH-oxidase subunit p47^phox^ [128].

In human studies, higher testosterone concentrations and FAI were associated with elevated levels of C-reactive protein in mid-life, early, and late postmenopausal women [129–132]. Others have reported no association in women aged 18–77 [133], and an inverse correlation between total testosterone and C-reactive protein in mid-life women and postmenopausal women on chronic hormone therapy [130, 134]. Whether testosterone administration in women alters the oxidative stress and inflammatory environment is unknown.

## Testosterone regulation of vascular adaptations to exercise

Although exercise is a powerful anti-aging strategy for vascular health, evidence suggests sex specificity in vascular adaptations to exercise training in older adults, particularly endothelial function. Endurance exercise has been shown to improve endothelial function in older men [135–137], but not older women [135, 138–140]. We recently demonstrated improvements in endothelial function with endurance exercise training in postmenopausal women treated with estradiol, but not in those treated with placebo, suggesting estradiol may have a key role in vascular adaptations to exercise in women [139].

The importance of testosterone to vascular adaptations to exercise in older men is not clear, but there is some evidence to suggest that it may be important. In men undergoing ADT for prostate cancer, 12 weeks of thrice-weekly endurance and resistance training improved endothelial function compared to controls, although differences were not maintained at 24 weeks [141]. A pilot study of diet plus exercise with or without testosterone supplementation in severely obese, hypogonadal men reported a significant improvement in endothelial function in the group receiving testosterone compared to diet plus exercise alone after 54 weeks [142]. These improvements were not maintained during a 24-week period of diet plus exercise alone. Finally, we demonstrated improvement in endothelial function with 12 months of progressive resistance training in healthy older men with borderline or low testosterone levels randomized to testosterone supplementation targeting either a lower range or usual range, but not those receiving placebo [85]. Mechanisms underlying this apparent synergy between testosterone and exercise in men may include common intracellular signaling pathways that promote NO release or modulation of gene expression in endothelial cells. It is also possible that androgens enhance vascular adaptations to exercise via conversion to estrogens, which then counteract oxidative and inflammatory damage.

Even less is known about how androgens influence vascular adaptations to exercise in women. To our knowledge, the only available evidence is from studies conducted in women with PCOS. Both macro- and microvascular endothelial functions have been shown to improve with moderate-intensity exercise training in this population, independent of changes in testosterone levels [143, 144]. In contrast, in a study of overweight or obese women with PCOS who were randomized to 20 weeks of diet, diet plus aerobic exercise, or diet plus combined aerobic and resistance exercise, endothelial function improved in all groups and was correlated with reductions in testosterone and FAI [145]. Importantly, this secondary analysis was underpowered to detect differences between groups and also used circulating markers of endothelial function (asymmetric dimethylarginine, plasminogen activator inhibitor-1, intra-cellular adhesion molecule-1, and vascular cell adhesion molecule-1), as opposed to measuring macro- or microvascular endothelial function. We are not aware of any studies that have examined the effects of androgens on the vascular response to exercise in healthy women or women with low testosterone.

## Summary

Vascular aging, featuring large elastic arterial stiffening and endothelial dysfunction, is thought to provide the milieu in which vascular diseases can flourish by combining with other pathophysiological CVD risk factors, creating a potentially lethal age-disease interaction. As such, elucidating mechanisms contributing to increased risk are needed for further development of strategies that attenuate or prevent the age-related changes in vascular function. Sex differences in vascular aging have been attributed to sex differences in gonadal aging and sex hormones. Although most of the literature on sex differences in vascular aging has focused on the effects of estrogen deficiency associated with menopause, little attention has been paid to the modulatory influence that declines in testosterone may contribute to vascular aging in women and men. Overall, available data support an association between lower levels of testosterone and endothelial function and increased arterial stiffness in men (Table 1). However, any effect of testosterone on vascular aging may be dependent on multiple factors including age, disease status, degree of testosterone deficiency, and form of testosterone supplementation, as well as effects from changes in other vasoactive hormones, such as estrogens. In contrast, the available data investigating the association of testosterone and vascular aging in women are more variable (Table 1). Moreover, the safety of long-term testosterone therapy on cardiovascular health has not been established. Thus, other strategies such as regular exercise are promoted as anti-aging interventions to target vascular aging in older adults. However, declines in gonadal function may also diminish beneficial endothelial adaptations to exercise training. The very limited data on the role of androgens in vascular adaptations to exercise suggest that testosterone may enhance improvements in endothelial function with exercise training in men with low levels of testosterone. Additional research is needed to further explore the effects of androgens on vascular adaptations to exercise in both men and women, as well as to explore underlying mechanisms for potential androgenic effects. Collectively, an improved understanding of the impact of declines in sex hormones, including androgens, on the aging vasculature at the cellular and systemic levels is needed to inform future sex-specific therapies for the prevention of CVD.

**Table 1:** Effects of endogenous low testosterone and testosterone supplementation on vascular aging and cardiovascular disease risk

Women	Endogenous low total testosterone		Testosterone supplementation	
	Perimenopausal	Postmenopausal	Perimenopausal	Postmenopausal
Cardiovascular disease risk	--	↑	--	--
Endothelial function				
Brachial artery FMD	↓	↑	--	↑
Digital peripheral artery tonomety	--	--	--	--
Arterial stiffness				
Pulse wave velocity	↔	↔	--	--
Augmentation index	↔	↔	--	--
Arterial compliance	↔	↔	--	--
Men	Endogenous low total testosterone		Testosterone supplementation	
	Middle age	Older	Middle age	Older
Cardiovascular disease risk	↑	↑	↑	↑↔
Endothelial function				
Brachial artery FMD	↓↑	↓↑	↔	↔↑
Digital peripheral artery tonomety	↓↑	↓↑	↔	↔
Arterial stiffness				
Pulse wave velocity	↑	↑	↓	↓
Augmentation index	↑	↑	↓	↓
Arterial compliance	↓	↓	↑	↑

-- no data available, ↑ elevated, ↓ reduced, ↔ no effect

## Data Availability

Data sharing is not applicable to this article as no datasets were generated or analyzed during the current study.

## Abbreviations

ADT: Androgen deprivation therapy
CEE: Conjugated equine estrogen
cf-PWV: Carotid-femoral pulse wave velocity
CHD: Coronary heart disease
CVD: Cardiovascular disease
DHEA: Dehydroepiandrosterone
DHT: Dihydrotesterone
eNOS: Endothelial nitric oxide synthase
FAI: Free Androgen Index
FMD: Flow-mediated dilation
FSH: Follicle-stimulating hormone
FTI: Free Testosterone Index
GnRH: Gonadotropin-releasing hormone
HSDD: Hypoactive sexual desire disorder
LC-MS/MS: Liquid chromatography-tandem mass spectrometry
LH: Luteinizing hormone
MESA: Multi-Ethnic Study of Atherosclerosis
NO: Nitric oxide
OVX: Ovariectomized
PCOS: Polycystic ovary syndrome
PWV: Pulse wave velocity
SHBG: Sex hormone-binding globulin
TNFα: Tumor necrosis factor-alpha
